# Hypoxia-Inducible Factor 1-Alpha Gene Polymorphisms Impact Risk of Severespectrum Hypertensive Disorders of Pregnancy: A Case-Control Study

**DOI:** 10.1007/s43032-025-01835-5

**Published:** 2025-03-14

**Authors:** Claire Baldauf, Chen Wei, Trevor A. Pickering, Brendan Grubbs, Håkon Gjessing, Melissa L. Wilson

**Affiliations:** 1 Keck School of Medicine, Department of Pediatrics, University of Southern California, Los Angeles, CA USA; 2 Children’s Hospital Los Angeles, Los Angeles, CA USA; 3 Keck School of Medicine, Department of Population and Public Health Sciences, University of Southern California, Los Angeles, CA USA; 4 Keck School of Medicine, Department of Obstetrics and Gynecology, University of Southern California, Los Angeles, CA USA; 5 Center for Fertility and Health, Norwegian Institute of Public Health, Oslo, Norway

**Keywords:** Allele, Genotype, *Haplin*, HELLP syndrome, Pre-eclampsia, Single nucleotide polymorphism

## Abstract

Hypoxia-inducible factor 1-alpha (HIF-1α) regulates cellular responses to hypoxia. Overexpression of HIF-1α is associated with abnormal placental trophoblast invasion and hypertensive disorders of pregnancy. We evaluated the putative association between polymorphisms and haplotypes in parental and child HIF-1α genes and the risk of severe-spectrum hypertensive disorders of pregnancy. Case (*N* = 179) and control (*N* = 34) mother-father-child triads were recruited by an internet-based method. Cases were defined as HELLP (Hemolysis, Elevated Liver enzymes and Low Platelets) syndrome or pre-eclampsia with severe features. Four HIF-1α single nucleotide polymorphisms were genotyped: rs4902080, rs2057492, rs11549465, rs10144958. Relative risks and 95% confidence intervals were estimated using log-linear free response models, adjusting for correlation between familial genotypes. Relative risk of severe-spectrum hypertensive disorder of pregnancy was increased with double-dose carriage of the T allele for SNP rs4902080 in both mother [RR 6.96, *p* = 0.028] and child [RR 5.77, *p* = 0.031]. Child double-dose of the T allele for SNP rs10144958 [RR 5.52, *p* = 0.047] also increased risk. The heterozygous genotype (CT) for SNPs rs2057482 and rs11549465 was protective against hypertensive disorders of pregnancy when carried by mother [rs2057482: RR 0.34, *p* < 0.001; rs11549465: RR 0.23, *p* < 0.001] or child [rs2057482: RR 0.44, *p* < 0.001; rs11549465: RR 0.31, *p* < 0.001]. A single copy of the C-c-c-G haplotype (rs4902080-rs2057482-rs11549465-rs10144958, *N* = 147), conferred decreased risk versus the C-T-T-G haplotype in mother [RR 0.28, *p* < 0.001] and child [RR 0.36, *p* < 0.001]. No parent-of-origin effects were seen. We conclude that polymorphism changes and haplotypes in the HIF-1α gene of mothers, fathers, and children are associated with risk for severe-spectrum hypertensive disorders of pregnancy.

**Supplementary Information:**

The online version contains supplementary material available at 10.1007/s43032-025-01835-5.

## Introduction

Hypertensive Disorders of Pregnancy (HDP) occur in 3–5% of pregnant women in the U.S [1, 2]. Short-term complications include maternal multi-organ dysfunction, hypertension, and seizures [3, 4]. Newborns are at increased risk for fetal growth-restriction, prematurity, and fetal demise [5, 6]. Maternal survivors of HDP have increased long-term risk of cardiovascular disease [3, 7, 8], diabetes mellitus [9], and all cause premature mortality (death prior to age 70 years) [10]. Long term sequelae for children include neurodevelopmental disorders [11–13], cerebral palsy (2-3X risk) [14, 15], and early onset metabolic syndrome [16, 17].

The pathogenesis of HDP remains unclear. Moreover, HDP is not one disease with one pathophysiology but comes in various clinical forms that appear mechanistically heterogenous. Shallow trophoblast invasion is associated with HDP [18], along with abnormal angiogenesis at the feto-maternal interface resulting in uteroplacental vascular resistance and chronic fetal hypoxia [19]. Thus, we focus here on genetic predisposition toward HDP by examining a gene known to be crucial for trophoblastic invasion and placental angiogenesis, HIF-1α.

HIF-1α is stabilized under hypoxic conditions and acts as a potent transcription factor [20, 21]. It is overexpressed in preeclamptic placentae [22–24] and involved in regulating downstream gene expression in response to changes in cellular oxygen tension [25–27]. In addition to its role in regulating downstream genes, HIF-1α protein suppresses trophoblast invasion and angiogenesis [28] through regulation of pro-angiogenic factors including vascular endothelial growth factor (VEGF) [29], soluble fms-related tyrosine kinase 1 (sFLT1) [30], transforming growth factor beta-3 (TGFβ3) [31, 32], and Placental Growth Factor 1 (PIGF1) [33], among others. HIF-1α also plays a role in placental cell fate [34].

HIF-1α gene polymorphisms are associated with a wide-spectrum of human diseases [35–42], including cardiovascular and neoplastic processes. Polymorphisms in HIF-1α, particularly the T allele in rs11549465 have been found to be associated with early onset HDP [24, 43].

Current literature examines primarily maternal susceptibility associated with specific single nucleotide polymorphisms (SNPs), although it would be expected that placental/fetal genotype is also an important factor. Imprinting and parent-of-origin effects [44] of the HIF-1α gene have not yet been determined and may also contribute to HDP risk. The primary aim of this study is to evaluate the putative association between HIF 1-α polymorphisms and haplotypes in all triad members and risk of severe-spectrum HDP. Secondarily, we evaluate the effects of a single-dose vs. double-dose of the variant allele and parent-of-origin effects.

## Materials and methods

### Subjects

This triad-based (mother-father-baby) pilot case-control study included 213 triads (179 cases and 34 controls). Cases were women with self-identified HELLP syndrome who were subsequently verified by chart abstraction (71% had charts available for verification). All unverified cases were considered to have severe-spectrum HDP since all reviewed cases met at least that definition. Only the index child was included when samples were available for more than one child. Participants were recruited online from one of two HELLP-focused websites (www.hellpsyndromesociety.org or https://www.facebook.com/pages/Hellp-Syndrome-Research-at-USC/163745723652843). Cases were asked to refer friends to the research team as controls if they had had a child within five years of the index pregnancy and had an unaffected pregnancy.

All study participation occurred via email or the postal service. Interested women contacted the Principal Investigator (MLW) to learn more. Participants were mailed consent forms, questionnaires, and saliva-based DNA collection kits. Paid self-addressed envelopes were included in study packets. Samples from mothers, fathers, and children were included in this study.

Exclusion criteria was maternal age less than 18 years. University of Southern California (USC) Health Science Campus Institutional Review Board (HS-06-00111) approved the study. Informed consent was obtained for all study participants providing samples and/or questionnaire data.

### Case Definition

Cases were classified as HELLP syndrome if medical records confirmed the following criteria after 20 weeks gestation: hemolysis (schistocytes, burr cells, or LDH > 600); elevated liver enzymes (AST > 70 and/or ALT > 70); and thrombocytopenia (platelets < 100 K). Participants with proteinuria ≥ 500 mg/dL in 24 h plus the presence of one or more severe features as defined by the American College of Obstetrics and Gynecology practice bulletin (No. 222) [45] were defined as having pre-eclampsia with severe features.

Among the 179 HDP cases, 28% were classified as HELLP syndrome and the remainder as pre-eclampsia with severe features.

### Data Abstraction

Medical records for cases were requested from delivery hospitals and obstetricians providing prenatal care. An investigator (MLW) reviewed records to confirm each diagnosis using a standardized data abstraction form that included information about obstetric history, prenatal visits, and delivery.

### Questionnaire

All participants completed a standardized risk factor questionnaire about their medical history, reproductive and sexual history, family history, and the affected pregnancy.

### Selection of Gene Polymorphisms

We selected four single nucleotide polymorphisms (SNP) within the HIF-1$\:\alpha\:$ gene: rs4902080, rs2057482, rs11549465, and rs10144958. SNP details are provided in Table 1. Each SNP exhibited at least one of the criteria below:

**Table 1:** HIF-1$\:\varvec{a}$ single nucleotide polymorphisms (SNPs) examined

SNP (rs#)	Position	Consequence	Allele change	Synonymous vs. nonsynonymous	Expected prevalence of minor allele^b^	Significant association
rs4902080	chr14:61741287 (GRCh38.p14)	Intron Variant	C > T	Not applicable	4.1%	None
rs2057482	chr14:61747130 (GRCh38.p14)	3-Prime UTR Variant^a^	C > T	Not applicable	15.2%	Mediation of lipids in myocardial infarction [41] Perimenopausal CAD [42]
rs11549465	chr14:61740839 (GRCh38.p14)	Missense Variant	C > T	missense variation nonsynonymous (Pro582Ser)	11%	Pre-eclampsia [24, 43] Immune thrombocytopenia [36] Lung cancer [73] Digestive cancer [74] Late onset Parkinson’s disease [39] Knee osteoarthritis [40]
rs10144958	chr14:61691980 (GRCh38.p14)	Intron Variant	G > T	Not Applicable	4.7%	None

^a^Other locations for this SNP are possible as more information about its gene structure becomes available

^b^Prevalence estimates from dbSNP from the National Library of Medicine

1. Known to be functional, based on published functional data.
2. Associated with an outcome in one or more peer-review publications.
3. Located in a coding region of the gene and results in a non-synonymous amino acid substitution.
4. Located in a non-coding region or regulatory region of the gene.
5. Located in an intronic region, which is evolutionarily conserved between placental mammals and humans.

### Sample Collection

DNA samples were collected via buccal swabs (21%) or saliva (79%) (DNA Genotek, Ottawa, Canada). The DNA sampling method did not affect the genotyping failure rate. Samples obtained from buccal swabs were extracted using QIAamp DNA Mini kits, per the manufacturer’s protocol (Qiagen, Valencia, CA). Saliva samples were extracted using ethanol precipitation, per the manufacturer’s protocol (DNA Genotek, Ottawa, Canada). Genotyping utilized TaqMan assays (7900HT Sequence Detection System, Applied Biosystems, Foster City, California, USA). A detailed protocol with laboratory techniques have been described previously [46].

### Statistical Analyses

Demographic and clinical characteristics are presented as mean ± standard deviation or median (inter quartile range, IQR) for continuous numeric variables and count with frequency for categorical variables, stratified according to case or control status. We used a log-linear method, implemented in the R package *Haplin* [47] to analyze case parent–triad data. Weinberg et al. described this log-linear method as based on a maximum likelihood of case-parent-triad data, with stratification of the parental mating type [48]. *Haplin* implements log-linear models to estimate relative risks (RR) with 95% confidence intervals [47, 49]. It estimates effects for single and double alleles, with free-response and multiplicative dose-response models and derives likelihood ratio $\:{X}^{2}\:$tests of association [49]. The unknown phase of haplotypes and missing samples (mother, father or child) were accounted for by the Expectation-Maximization (EM) algorithm, in *Haplin* [47, 50].

We estimated single- and double-dose effects for mothers and babies. We also evaluated free response and multiplicative models, assessed haplotypes, and evaluated parent-of-origin effects. Multiplicative models were not significant, and as this model assumes alleles are functionally linked, it cannot estimate independent effects of single-dose vs. double-dose of the variant allele [49]. Sensitivity analysis examined the impact of imputed genotypes by re-running the analysis excluding imputed data. We found no substantive differences and therefore present the results with imputations to preserve sample size. All SNPs had less than 10% imputed data (Online Resource Supplementary Table 1).

The most common allele and haplotype in our cohort were used as referent groups. The effects of single-dose (heterozygous) and double-dose (homozygous) variant alleles and haplotypes were estimated. Rare haplotypes (frequency < 5%) were excluded from analysis, resulting in a comparison of two haplotypes. A two-sided significance level of α = 0.05 was used. Analyses were conducted using R software (version 4.0.2).

Further details on the *Haplin* package, including manual in PDF format are available on the CRAN website [51]. This study was conceived as a pilot study and thus, no a priori power calculation was made. This study was conceived as a pilot study and thus, no a priori power calculation was made. Given the minor allele prevalence (listed in Table 1) for each individual SNP in our study, we calculate that with 179 case triads and 34 control triads, we have 80% power to detect relative risks between 1.5 and 2.3, assuming a Type I error rate of 5% (estimate calculated using Haplin, in R).

## Results

### Demographics

Maternal demographics and clinical characteristics are presented in Table 2. The average maternal age among controls and cases were similar (32.3 ± 3.9 and 31.1 ± 4.0 years) respectively. Almost all participants were white (cases 98.4%, controls 100%). The gestational age at delivery was lower in cases than controls (33.0 ± 4.5 vs. 39.6 ± 1.7 weeks), respectively. The median birth weight for case newborns (1942.5 g (IQR 1181.5, 2786.5)) was consistent with the low mean gestational age. A higher proportion of cases were nulliparous (88.4%) versus controls (50%). Case laboratory values demonstrated elevated lactate dehydrogenase (601.5 U/L (IQR 337.0, 1306.3)), aspartate aminotransferase (253.0 U/L (IQR 115.3, 447.5)), and alanine aminotransferase (204.0 U/L (IQR 117.0, 354.0)), with thrombocytopenia (platelets 60.0 10^9^/L (IQR 37.0, 99.5)).

**Table 2:** Maternal demographics and clinical characteristics

Variable^a^	*N* ^b^	Control (Total *N* = 34)	*N* ^b^	Case (Total *N* = 179)
Age (years)	29	32.3 ± 3.9	131	31.1 ± 4.0
White race (%)	30	30.0 (100.0)	128	126.0 (98.4)
Gestational age at delivery (weeks)	22	39.6 ± 1.7	125	33.0 ± 4.5
Pre-pregnancy weight (lbs)			108	149.9 ± 33.0
Nulliparity (%)	26		129	
Nulliparous		13.0 (50)		114.0 (88.4)
Parous		13.0 (50)		15.0 (11.6)
Parity	24		120	
0		11(45.9)		107.0 (89.1)
1		8 (33.3)		8.0 (6.7)
2 or more		5 (20.8)		5 (4.2)
Gravidity	24		121	
1		11.0 (45.8)		91.0 (75.2)
2		6.0(25.0)		21.0 (17.4)
3		4.0(16.7)		5.0 (4.1)
4 or more		3.0(12.5)		4.0 (3.3)
Status	NA		179	
Pre-eclampsia with severe features				129 (72.1)
HELLP Syndrome				50 (27.9)
Lactate dehydrogenase, U/L	NA		64	601.5 (337.0, 1306.3)
Bilirubin, mg/dL	NA		84	0.9 (0.5, 2.0)
Aspartate aminotransferase, U/L	NA		112	253.0 (115.3, 447.5)
Alanine aminotransferase, U/L	NA		105	204.0 (117.0, 354.0)
Creatinine(mg/dL)	NA		96	0.8 (0.7, 1.0))
Platelet count, 10^9^/L	NA		113	60.0 (37.0, 99.5)
Birthweight, grams	NA		106	1942.5 (1181.5, 2786.5)
Fetal growth restriction (%)	NA		118	11.0 (9.3)
Gestational diabetes (%)	NA		118	8.0 (6.8)

^a^Numeric variables are presented as mean (SD) or median (IQR) while categorical variables are presented as count (frequency)

^b^N of available subject data for each variable upon which statistic is reported. Of cases, 71% (127/179) had charts available for data abstraction. Control charts were not abstracted

### Polymorphisms in the Maternal and Child HIF-1α Gene and Risk of HDP

Table 3 presents the maternal and fetal effects of each HIF-1$\:a$ SNP. Bold numbers indicate statistical significance at alpha ≤ 0.05. Both single and double doses were examined in a free response model; multiplicative models were not significant.

**Table 3:** Maternal and child carriage of HIF-1$\:\varvec{a}$ polymorphisms and risk of HELLP syndrome and Pre-eclampsia with severe features

SNP, minor allele	Maternal					Child				
	Minor Allele Frequency (%)	Single dose RR (95% CI)	*P*-value	Double dose RR (95% CI)	*P*-value	Minor Allele Frequency (%)	Single dose RR (95% CI)	*P*-value	Double dose RR (95% CI)	*P*-value
rs4902080, T	4.2	1.29 (0.63, 2.62)	0.49	**6.96** **(1.24**,** 38.6)**	**0.028**	4.7	0.73 (0.34, 1.57)	0.425	**5.77** **(1.18**,** 29.20)**	**0.031**
rs2057482, C	40.8	**0.34** **(0.28**,** 0.54)**	**< 0.001**	1.23 (0.66, 2.28)	0.505	39.2	**0.44** **(0.28**,** 0.68)**	**< 0.001**	1.08 (0.56, 2.08)	0.827
rs11549465, C	40.9	**0.23** **(0.14**,** 0.38)**	**< 0.001**	1.05 (0.54, 2.04)	0.891	38.5	**0.31** **(0.19**,** 0.50)**	**< 0.001**	1.29 (0.66, 2.57)	0.463
rs10144958, T	5.0	0.72 (0.35, 1.45)	0.358	1.91 (0.23, 17.50)	0.549	5.0	1.06 (0.51, 2.17)	0.875	**5.52** **(1.04**,** 30.70)**	**0.047**

#### SNP rs4902080

We found a statistically significant increase in HDP risk with double dose carriage of the T allele for SNP rs4902080 for both mother [RR 6.96, CI (1.24, 38.6), *p* = 0.028] and child [RR 5.77, CI (1.18, 29.20), *p* = 0.031],

*SNP rs2057482: We found* the CT genotype (heterozygotes) for SNP rs2057482 in both maternal [RR 0.34, CI (0.28, 0.54), *p* < 0.001] and child [RR 0.44, CI (0.28, 0.68), *p* < 0.001] subjects was protective against HDP.

#### SNP rs11549465

The CT genotype for SNP rs11549465 in mother [RR 0.23, CI (0.14, 0.38), *p* < 0.001] and child [RR 0.31, CI (0.19, 0.50), *p* < 0.001] was protective against HDP.

#### SNP rs10144958

Double dose carriage by the child of the T allele in SNP rs10144958 significantly increased risk of HDP [RR 5.52, CI(1.04–30.70, *p* = 0.047].

### Haplotype and Risk of HDP

The association between maternal and child haplotypes and HDP (rs4902080- rs2057482- rs11549465- rs10144958; *N* = 147) is presented in Table 4. For both maternal [RR 0.28, CI (0.16, 0.50), *p* < 0.001] and child [RR 0.36, CI (0.20, 0.63), *p* < 0.001] samples, we found a reduced risk of HDP among carriers of the C-c-c-G versus C-T-T-G haplotype in the single, but not double-dose model.

**Table 4:** Maternal and child carriage of HIF-1α haplotypes and risk of HELLP syndrome and Pre-eclampsia with severe features

Haplotype	Frequency (%)	Maternal				Child			
		Single dose RR (95% CI)	*P*-value	Double dose RR (95% CI)	*P*-value	Single dose RR (95% CI)	*P*-value	Double dose RR (95% CI)	*P*-value
rs4902080- rs2057482- rs11549465- rs10144958 (*N* = 147)									
C-T-T-G	58.9	REF		REF	0.348	REF		REF	0.731
C-c-c-G	41.1	**0.28** **(0.16**,** 0.50)**	**< 0.001**	1.2 (0.55, 2.60)	0.648	**0.36** **(0.20**,** 0.63)**	**< 0.001**	1.13 (0.51, 2.55)	0.765

### Parent-of-Origin Effect

No parent-of-origin effect was detected in the evaluated SNPs, or in the haplotype analysis (Online Resource Supplementary Tables 2 and 3).

## Discussion

### Principal Findings

We found HIF-1α gene SNPs significantly impact risk for HELLP syndrome or pre-eclampsia with severe features. All triad members affect the risk profile, with no observable parent-of-origin effects.

### SNP rs1154965 and Risk of Hypertensive Disorders of Pregnancy

Consistent with our findings, Andraweera et al. reported a protective effect against early-onset pre-eclampsia in women with a single T allele (heterozygotes) for SNP rs1154965 [43]. This was found *after stratifying* by early vs. late onset disease. No early-onset pre-eclampsia cases had the TT genotype thus it was not examined; in our study, the TT genotype was not protective. Three additional studies in Finnish [52], Mexican [53], and Korean [54] populations did not find an association between SNP rs1154965 and pre-eclampsia, however they did *not* stratify early versus late onset disease. This could explain the difference in results, as early and late-onset pre-eclampsia are increasingly recognized as pathophysiologically distinct disorders [55, 56]. Given the importance of HIF-1α in trophoblast cell invasion during placentation, it follows that early onset pre-eclampsia is more affected by these SNP variants. Contrary to our results, Harati-Sadegh et al. found that for SNP rs1154965, carriage of at least one T allele increased risk for pre-eclampsia versus the CC genotype [24]. This may reflect the less severe pre-eclamptic population in their study versus our severe spectrum HDP cohort.

### Potential Mechanisms for HIF-1α Variants rs1154965 and rs2057482 and their Relationship To HDP

Previous examination of *rs1154965* found a double-dose T allele associated with increased HIF-1$\:\alpha\:$ mRNA expression [24], suggesting it drives increased HIF-1α transcription for TT homozygotes. Interestingly, in a study examining adult cardiovascular disease risk factors, Hlatky et al. found the T allele in both homo and heterozygotes for SNP rs1154965 and rs2057482 have lower HIF-1α protein transcriptional *activity* than wild type (CC) at comparable HIF-1α mRNA levels [57]. Compared to heterozygotes, TT homozygotes had the largest effects. Increased mRNA levels in conjunction with lower protein activity may point to the T allele creating a less *functional* protein and stimulating positive feedback for HIF-1α mRNA production during hypoxic conditions. Indeed, this may explain previous studies that find increased HIF-1α *expression* in early-onset pre-eclamptic placentae [58–60], despite pathologic findings (shallow extravillous trophoblast invasion) suggesting abnormal HIF-1α pathway *function*. Replication of these molecular findings is warranted to elucidate how SNP variants affect phenotypic change.

In studies of HIF-1α SNPs in coronary artery disease, the TT genotype of rs1154965 and rs2057482 was protective [57, 61]. In our study however, we only found a protective effect of the CT genotype. Though both vascular, these are differing physiologies and tissue microenvironments; for HDP the T allele effects on HIF-1α activity may be most protective in mediation. Alternatively, there may be a heterozygote advantage effect of the CT genotype for these HIF-1α loci in the context of pregnancy, such as the case for phenylketonuria maternal heterozygotes and decreased fetal loss in pregnancy [62, 63].

### Associations for SNPs rs4902080 and rs10144958

We did not find studies associating rs4902080 and rs10144958 with a particular disease phenotype or molecular change in HIF-1α expression. Our finding that a double dose of the T allele increases risk for HDP significantly for maternal and/or fetal carriage warrants reproduction in future studies.

### Clinical Implications

Clinical studies have shown that women with a family history of pre-eclampsia are at increased risk for the disorder, especially early onset/severe disease, with paternal genetics also playing a role [64–67]. Our findings bear out that both parental genotypes affect risk, in the same direction and with similar magnitude. Of the triad, the fetal genetic contribution to HDP appears most important with genotype affecting HDP risk for all examined SNPs. Given the pathogenesis of severe spectrum HDP is likely in first trimester, potential HIF-1α pathway-based interventions, are most likely to be successful early in pregnancy.

Personalized risk tools for severe spectrum hypertensive disorders of pregnancy are being investigated such as utilizing microRNA [68] and multi-variable machine learning [69]. Variants for genes involved in placental development, including HIF-1α may add valuable information in targeted risk stratification approaches. With the advent of non-invasive pre-natal testing, future inclusion of genetic associated pregnancy risks is foreseeable.

Further research into the effects of HIF-1α SNP and haplotype variations in a larger, more diverse patient population will be necessary before this data can be utilized in clinical practice.

### Research Implications

Future comparison of the molecular effects of HIF-1α SNP variants is warranted, for both it and its myriad downstream effectors. Longitudinal evaluation will help inform how SNPs affect HIF-1α function during placentation versus later in pregnancy, especially regarding early versus late-onset placental disease. Tying molecular data to histopathology such as anomalous uterine invasion of extravillous trophoblast and spiral artery changes will help speed translation of risk stratification and pathophysiology-based interventions to practice.

### Strengths and Limitations

This study has several limitations. The small sample size, particularly control triads, only permitted detection of relative risks 1.5 and higher, where lower values would still be clinically significant. Confidence intervals for double dose effects were wider than single, likely due to lower double dose carriage sample size in these groups and thus potential inability to reach statistical significance (Type II error).A small number of participants carrying a double dose of the variant can make estimates of double versus single-dose unstable [70]. For some variants, the single dose allele altered risk of HDP whereas the double dose did not. For instance, in rs11549465 and rs2057482, one T allele was protective but two was not.

Two (rs11549465 and rs2057482) of the four SNPs investigated showed deviations from Hardy-Weinberg equilibrium (HWE). While deviations from HWE may indicate mating selection, inbreeding, non-paternity, or other factors [71], they can also be caused by maternal-fetal interactions [72] since such interactions increase the complexity of the genetic model and can lead to inaccurate estimation of variant frequency and distribution. Interestingly, maternal-fetal interactions may also explain why mothers who were heterozygous at these SNPs might be at lower risk; their genotype includes a copy of the paternal allele transmitted to the child, potentially reducing fetal-maternal mismatch. As we would expect the maternal and fetal genomes to interact in a genomically complex condition such as pregnancy, maternal-fetal interaction may thus explain some of the unusual patterns of allelic distributions seen in our study.

We could only obtain records to confirm 71% of case diagnoses, and control pregnancy records were not reviewed. Thus, unconfirmed cases who were classified as severe-spectrum HDP may have been cases of HELLP syndrome and thus, misclassified. However, since all severe cases were combined in this analysis, there should have been no substantive impact on our results. If some women with HDP were inadvertently included in the control group, the resulting relative risk would be attenuated toward the null. Additionally, our population was almost entirely White, thereby limiting the generalizability of our results to other racial and ethnic populations. Last, we recruited participants online, and all affected women do not have equal internet access. Thus, our population likely had a higher socioeconomic status than the overall population of women with HDP.

This study has several strengths. First, it encompasses the assessment of mother-father-baby triads, enabling the estimation of genetic influences for each family member and allowing for the examination of parent-of-origin effects. The triad-based study design improves statistical power as does inclusion of both controls and cases [75]. While we were unable to obtain records for all cases, the fact that all verifiable cases were correctly categorized helps support the accuracy of self-reported case vs. control status. Future studies should include a larger sample size from a more diverse population and verification of case-control status for all participants.

## Conclusion

Polymorphisms within the HIF-1α gene may be associated with risk of severe-spectrum HDP in both parent and child samples. Our findings suggest a possible maternal-fetal interaction, though our sample size was insufficient to model this effect. No parent-of-origin effects were observed.

## Electronic Supplementary Material

Below is the link to the electronic supplementary material.

Supplementary Material 1
