# Supplementary material for: Hypoxia-Inducible Factor 1-Alpha Gene Polymorphisms Impact Risk of Severespectrum Hypertensive Disorders of Pregnancy: A Case-Control Study
Source: Reprod Sci. 2025 Mar 14;32(4):993–1002. doi: 10.1007/s43032-025-01835-5 (PMC11978723; doi:10.1007/s43032-025-01835-5)
Supplement: Supplementary file 1 — Supplementary Material 1 [file 43032_2025_1835_MOESM1_ESM.pdf]

Proposal #HS-06-00111

University of Southern California Institutional Review Board
1640 Marengo Street, Suite 700
Los Angeles, California 90033-9269
Telephone: (323) 442-0114
Fax: (323) 224-8389
Email: irb@usc.edu

Date: Sep 23, 2020, 04:04pm
To: Melissa Wilson
PREVENTIVE MEDICINE

From: University of Southern California Institutional Review Board
1640 Marengo Street, Suite 700
Los Angeles, California 90033-9269
(323) 442-0114

TITLE OF PROPOSAL:
Genetic Susceptibilty to HELLP Syndrome and Severe Preeclampsia
Continuing Review: HS-06-00111-CR016 (Continuing Review 2019-2020)

Action Date: Action Taken:
Committee: Institutional Review Board Chairman
Note: The University of Southern California Institutional Review Board (IRB) designee reviewed your continuing review and was **APPROVED on 9/23/2020**. The Continuing Review qualifies for expedited review according to 45 CFR 46.110 as Research Category 3, 5 & 7.

Per the revised common rule 45 CFR 46.109(f)(1)(i), this study is no longer subject to continuing review requirements.

The materials submitted and considered for review included:

1. Continuing Review Form, dated 9/23/2020

PRINCIPAL INVESTIGATOR RESPONSIBILITIES:

As the Principal Investigator, you are required to ensure that this research and the actions of all project personnel involved in conducting the study will conform with the research project and its modifications approved by the IRB; HHS regulations (45 CFR 46); International Conference on Harmonization Good Clinical Practice Consolidated Guideline; IRB Policies and Procedures and applicable state laws.

You must inform the IRB immediately if you become aware of any violations of HHS regulations (45 CFR 46), applicable state laws or IRB Policies and Procedures for the protection of human subjects.

Attachments:

This is an auto-generated email. Please do not respond directly to this message using the "reply" address. A response sent in this manner cannot be answered. If you have further questions, please contact your IRB Administrator or IRB/CCI office.

The contents of this email are confidential and intended for the specified recipients only. If you have received this email in error, please notify istar@usc.edu and delete this message.
